# 2477: Biofilms in wounds: Detection, individualizing treatment and monitoring response to therapy

**DOI:** 10.1017/cts.2017.233

**Published:** 2018-05-10

**Authors:** Petra Wilder-Smith, Janet Ajdaharian, Afarin Golabgir Anbarani, Jessica Ho, Karan Sahni, Richa Mittal, Eric Potma

**Affiliations:** Institute for Clinical and Translational Science, University of California, Irvine, CA, USA

## Abstract

OBJECTIVES/SPECIFIC AIMS: The specific objectives of this project are (1) identify, test, and validate the parameters for a simplified NLOM imaging probe that will provide specific research and point-of-care information on biofilm presence, therapeutic need and response of individual wounds to treatment. (2) Identify specific proteomic and metabolomic biomarkers of (i) wound susceptibility to infection, (ii) wound response to the most commonly used antibacterial measures in wounds, and (iii) establish criteria for more effective interventions. METHODS/STUDY POPULATION: First, optimal use parameters for NLOM including illumination, field of view, focal length, linear Versus concentric image acquisition, detection and filter wavelengths were identified. Parameters for evaluation included ease and speed of imaging, ability to map diagnostic criteria. Next, using the optimised NLOM imaging modality in bacterial biofilm isolates and subsequently a rabbit ear model of biofilm wound infection, proteomic and metabolomic biomarkers of susceptibility to infection were identified. The effects of 2 standard debridement and anti-infective treatments, polyvidone-iodine solution or cetrimide 15%+ chlorhexidine gluconate 1.5% were mapped in situ for up to 10 days using the NLOM probe. RESULTS/ANTICIPATED RESULTS: Using the novel custom NLOM probe, high resolution mapping of wound biofilm infection, as well as the underlying tissue was performed throughout the onset, development, treatment, and resolution of wound biofilm infection. Specific microbiological, microstructural, oxygenation, and pH parameters were mapped at defined surface and subsurface locations and time-points. Findings included the determination that some standard antimicrobial formulations provide a supportive environment for wound infection, and that micro-channels within the biofilm and their interface with the tissues serve as an important predictor and indicator of wound infection establishment, progression, and response. DISCUSSION/SIGNIFICANCE OF IMPACT: The novel multimodality in vivo NLOM imaging approach establishes an important tool for earlier and more specific diagnosis of wound infection risk, virulence, and invasiveness along with markers of successful treatment, and a simple clinical imaging tool for improving wound infection prevention and treatment.

